# Initial leucocytosis and other significant indicators of poor outcome in severe traumatic brain injury: an observational study

**DOI:** 10.1186/s41016-020-0185-4

**Published:** 2020-02-10

**Authors:** Kugan Vijian, Eu Gene Teo, Davendran Kanesen, Albert Sii Hieng Wong

**Affiliations:** Department of Neurosurgery, Sarawak General Hospital, Kuching, Sarawak Malaysia

**Keywords:** Leucocytosis, Severe traumatic brain injury, Outcome

## Abstract

**Background:**

Globally, severe traumatic brain injury (TBI) has been the principal cause of mortality among individuals aged 45 and below. The incidence of road traffic accidents in Malaysia is one of the highest in the world with thousands of victims sustaining severe disabilities. The aim of this study is to determine the association between leucocytosis and extended Glasgow Outcome Scale (GOSE) scores as well the relationship of other factors and the outcomes of severe TBI.

**Methods:**

This was a retrospective observational study. A total of 44 consecutive patients who were admitted to Sarawak General Hospital from January 1, 2018, to September 30, 2018, with severe TBI were included. Data were collected from discharge summaries and hospital medical records. Chi-square and *t* test were used. SPSS was employed.

**Results:**

Of a total of 44 patients with severe TBI, 18 patients (41%) died during the same admission. The mean age of patients was 37.1 years with 93.2% of affected patients being male. 56.9% of patients presented with a Glasgow Coma Scale (GCS) of 6 and less. A large percentage (86.3%) were discharged with a GOSE of less than 7. Older age and low admission GCS (6 and less) were significantly associated with poor GOSE scores on discharge and after 6 months (*p* < 0.05) on multivariate analysis. Leucocytosis on admission was also associated with poor outcomes where patients with higher total white counts on presentation attaining lower GOSE scores (*p* < 0.05).

**Conclusion:**

We concluded that leucocytosis was significantly associated with poor outcomes in severe TBI patients in addition to other factors such as advanced age and poor GCS on arrival.

## Background

Across the world, traumatic brain injury (TBI) has been estimated to affect 69 million individuals annually, exceeding the number of casualties seen in World War I [1, 2]. Young adults and the paediatric age group are seen to account for most deaths related to TBI [3]. Severe TBI, being the worst spectrum of the condition, significantly contributes to the health and economic burden of a nation. Malaysia, belonging to the South-East Asia region, has more than 50,000 trauma patients being admitted to the emergency department each year with Glasgow Coma Scale (GCS) of less than 8 [4]. Most of the patients with severe TBI are left with disabilities impeding their return to normal daily routines and self-care.

Research into the biochemical markers associated with TBI has been extensive to say the least. Levels of myelin basic protein (MBP), neuron-specific enolase (NSE), glial fibrillary acid protein (GFAP), and S100 are among the markers being studied in relation to TBI. However, their reliability as prognostic or severity indicators is still questionable due to the numerous factors affecting their release and serum concentrations [5]. Leucocytes, being acute phase reactants, respond to stress and injury similarly as how they would towards an infection. The inflammatory response triggered by a TBI has been hypothesised to involve activation of microglia, lymphocyte infiltration, and neutrophil margination [6]. The leucocyte count, an easy to acquire and fairly simple test, may also be raised by injuries to other organs or systems especially injuries associated with higher Injury Severity Score (ISS).

The Brain Trauma Foundation, which was founded in 1986, presented guidelines of early indicators which prognosticate outcomes in severe TBI. This includes GCS, age, and CT scan features to name a few [7]. Disability, being a major consequence of this condition, can be scored rather accurately with the Glasgow Outcome Scale (GOS) and the extended Glasgow Outcome Scale (GOSE). They serve as outcome measurements to numerous research on TBI and the factors contributing to its adverse sequelae.

The aim of this study is to determine the association between leucocytosis and GOSE scores as well the relationship of other factors and the outcomes of severe TBI.

## Methods

This was a retrospective observational study conducted in the Sarawak General Hospital. It is a 1005 bedded hospital and serves as a tertiary and referral centre for the state of Sarawak, Malaysia. It is also the largest hospital in the state.

### Sample population

This study involved 44 consecutive patients who sustained severe TBI over a period of 9 months which spans from the 1 January 2018 to 30 September 2018. All patients were Malaysian citizens of various ethnic backgrounds. All cases were admitted via the emergency department of Sarawak General Hospital and subsequently nursed in the neurosurgical high dependency unit.

All patients included in this study were between ages 12 to 78 and diagnosed with severe TBI, characterised by Glasgow coma scale (GCS) ≤ 8 on admission after adequate resuscitative efforts resulting from a non-penetrating head injury. Patients excluded from this study were as follows: (a) patients with polytrauma or Injury Severity Score (ISS) of more than 15, (b) patients with major multiorgan dysfunction, and (c) patients with suspected seizures or spontaneous intracranial haemorrhage preceding the TBI. No patients had any history of active or recent infections, and none were on steroids or other immunosuppressants. Two patients included in this study had diabetes mellitus. Both were on oral hypoglycaemic agents, and their blood sugar levels were within normal range during admission and subsequent hospitalisation.

Both groups of patients who were treated conservatively and with surgery were included. All patients were primarily managed by the neurosurgical team and were co-managed by the anaesthetic team upon disposition by the emergency department.

### Data collection and analysis

Patients’ medical records and operative notes were traced from the medical records office, discharge summaries, and operating theatre database. Socio-demographic details such as age, ethnicity, and gender in addition to details pertaining to TBI such as GCS on admission and discharge, type of TBI, predominant side and site of bleeding, presence of midline shift, type of surgery performed and ambulatory status on discharge, and patients’ GOSE score were collected and tabulated.

Patients and family members were then contacted to acquire information regarding their current clinical condition and GOSE scores. The data were tabulated in Microsoft Excel and analysed using the SPSS software version 20.0 (SPSS Inc., Chicago, IL). Frequency distribution table, bar charts, means, and percentage were used for descriptive data. Chi-square, independent sample *t* test, odds ratio, and 95% confidence interval were calculated. *p* values of < 0.05 were regarded as statistically significant.

### Endpoints

The primary endpoints were the patients’ GOSE at 6 months post-insult. The GOSE is a reliable tool that scores patients based on the degree of disability they suffer. The score ranges from 1 (dead) to 8 (full recovery with no problems relating to the injury).

## Results

The mean age of patients was 37.1 years old with the majority of patients being male (93.2%). Most of them presented with a GCS of 3 (27.3%), 7 (25%), and 8 (18.2%). More than half of the sample size presented with unequal or fixed pupils (52.2%). The type of bleeding varied among patients, and 45.5% patients had midline shift on initial CT scan. Surgery was conducted on 17 patients while the remaining were treated conservatively. The mean leucocyte count in our sample was 17.2 × 10^9^/L with predominant neutrophilia (85.3%) (Table 1).

**Table 1:** Socio-demographic and features of TBI

Variable	Frequency (%)
Age (mean ± SD)	37.1 ± 19.5
Gender	
Male	41 (93.2)
Female	3 (6.8)
Employment	
Working	21 (47.7)
Non-working	14 (31.8)
Student	9 (20.5)
Marital status	
Married	22 (50.0)
Non-married	22 (50.0)
GCS on admission	
8	8 (18.2)
7	11 (25.0)
6	5 (11.4)
5	5 (11.4)
4	3 (6.8)
3	12 (27.3)
Leucocyte count (mean ± SD)	17.2 ± 8.5 (10^9^/L)
Neutrophils (%)	85.3
Lymphocytes (%)	8.0
Monocytes (%)	6.7
Pupils	
Equal and reactive	21 (47.7)
Unequal	17 (38.6)
Fixed	6 (13.6)
Type of bleeding	
EDH	3 (6.8)
SDH	18 (40.9)
Intraparenchymal	20 (45.5)
IVH	2 (4.5)
SAH	6 (13.6)
DAI	13 (29.5)
Midline shift	20 (45.5)
Predominant side of bleeding	
Left side	20 (45.5)
Right side	13 (29.5)
Bilateral/no predominant side	11 (25.0)
Surgery	17 (38.6)

Among the patients who survived and were eventually discharged, 50% had a GCS of 15 while 42.3% of patients were discharged with a GCS of 11 and less. Eighteen patients died during admission (Table 2).

**Table 2:** Outcome of patients

Variable	Frequency (%)
GCS on discharge	
15	13 (50.0)
12–14	2 (7.7)
11 and below	11 (42.3)
Ambulation on discharge	
Bed bound	11 (38.5)
Wheelchair	8 (38.0)
Walking	8 (38.0)
Sacral sore	6 (20.0)
Pneumonia	11 (42.3)
Tracheostomy	12 (46.2)
GOSE score > 6 months	
7–8	9 (20.5)
5–6	7 (15.9)
3–4	10 (22.8)
1–2	18 (40.9)
Death after admission	18 (40.9)

Table 3 shows that significant association was found between leucocyte count on arrival with GOSE score > 6 months post-trauma. Higher leucocyte counts were significantly associated with GOSE scores of 6 or less (*p* < 0.05). Older age, unequal or fixed pupils, midline shift, and GCS of 6 or less were also significantly associated with low GOSE scores (*p* < 0.05) on univariate analysis. To eliminate the effects of confounding factors in this study, variables with a *p* < 0.05 in the univariate analysis were fitted into a logistic regression model. In the multivariate analysis, older age, GCS of 6 or less, and higher leucocyte counts were significantly associated with poor outcomes (Table 4).

**Table 3:** Univariate analysis of factors affecting outcome of severe TBI

Variable	Frequency of GOSE score at 6 months		*p* value
	7–8	< 7	
Age (mean)	24.8 ± 12.6	40.3 ± 19.9	0.01^
Gender			
Male	8 (89)	33 (94)	
Female	1 (11)	2 (6)	0.50
GCS ≤ 6 on arrival	2 (22)	23 (66)	
GCS > 6 on arrival	7 (78)	12 (34)	0.02*
Midline shift on initial CT	1 (11)	9 (53)	0.03*
Leucocyte count (mean) (10^9^/L)	12.0 ± 6.4	20 ± 8.4	0.03^
Pupils (fixed/unequal)	0 (0)	23 (66)	0.01*
Predominant side (Left)	6 (67)	6 (17)	0.11

*Chi-square

^*t* test

**Table 4:** Multivariate analysis

Variable	OR (95% CI)	*p* value
Age (mean)	0.94 (0.89–0.99)	0.04
GCS ≤ 6 on arrival	0.12 (0.02–0.78)	0.02
Midline shift on initial CT	9.0 (0.91–88.4)	0.06
Leucocyte count (mean) (10^9^/L)	0.71 (0.52–0.99)	0.04
Pupils (fixed/unequal)	1.00 (0.01–1.01)	0.98

Variables with *p* < 0.05 in the univariate analysis are fitted into the above logistic regression model

## Discussion

This retrospective study demonstrates that leucocytosis on the initial presentation of severe TBI patients was significantly associated with lower GOSE scores after 6 months of head injury. Older age group and poor GCS on admission were also significantly associated with GOSE scores of 6 and less based on multivariate analysis. Midline shifts on initial CT scan and pupillary changes showed significant association with poor outcomes in the univariate analysis.

The leucocyte count, being a routinely ordered investigation is performed for almost all patients sustaining trauma. This blood investigation can be done in districts as well as primary healthcare centres. Its interpretation requires no added knowledge or skill, and the results can even be available in minutes. Its levels, although affected by numerous factors, can still be deemed reliable if the investigation is performed on the earliest encounter and with gross patient selection. Theories on the series of events leading to the elevation of leucocyte count in the blood have revolved around the response mounted by microglia, neutrophils, and lymphocytes in relation to the extent of head injury [6]. Major intracranial injuries have been shown to have higher leucocyte counts, pertaining to the higher degree of the inflammatory cascade activation. Patients with more extensive and severe traumatic brain injuries are often associated with far worse outcomes, and they are often accompanied by a greater rise in leucocyte count [8].

The lack of resources and experience in using recently available and sophisticated biochemical markers to prognosticate outcomes is not the only drawback. These markers have been shown to demonstrate variability in sensitivity due to physiological releases, age and gender differences in levels, and changes in rate of clearance due to specific organ dysfunctions [5].

The mean age of patients being affected by severe TBI was 37.1 years old similar to previous published literature on severe TBI in Malaysia [9]. This was seen as adults were the highest users of motor-powered vehicles on the road and were therefore mostly implicated in road traffic accidents. A male preponderance is also seen in this study in line with other studies on head injury; however, a higher mortality rate in females was published by certain studies [9–11]. Older age groups were shown to have worse outcomes after sustaining severe TBI as studies have shown patients of younger age group to have better recovery and higher chances of returning to normal life and work in addition to a twofold rise in mortality rates in the elderly following a TBI [12, 13].

This study shows a rather scattered distribution of GCS on admission ranging from 3 to 8 with lower GCS on presentation, mainly 6 or less leading to poor outcomes. The importance of GCS as an indicator of severity has been repeatedly tested and proven. Nevertheless, accurate and early assessment remains important in utilising this score [14, 15]. The relationship between unequal/fixed pupils and midline shift on CT scan with poor outcomes is supported by class 1 evidences showing high positive predictive values for unfavourable outcomes in the presence of these features [16, 17].

## Conclusions

The prediction of an individual’s future recovery following a severe TBI is without question of important value. It guides rehabilitation planning and aids counselling of family members and carers. While the pursuit to obtain a single gold-standard marker in TBI might be within reach in the advent of recent research advancements, the significance of basic clinical assessments and lab values should not be disregarded.

## Data Availability

The datasets used and/or analysed during the current study are available from the corresponding author on reasonable request.

## References

[1] Dewan Michael C., Rattani Abbas, Gupta Saksham, Baticulon Ronnie E., Hung Ya-Ching, Punchak Maria, Agrawal Amit, Adeleye Amos O., Shrime Mark G., Rubiano Andrés M., Rosenfeld Jeffrey V., Park Kee B. (2019). Estimating the global incidence of traumatic brain injury. Journal of Neurosurgery.

[2] Nadège M. REPERES: World War I casualties- EN. CVCE. 2011.

[3] Moppett IK (2007). Traumatic brain injury: assessment, resuscitation and early management. Br J Anaesth..

[4] Ministry of Health Malaysia. National Trauma Database. January to 2007. Second report. Assessed on July 18 2019.

[5] Romner B (2000). Traumatic brain injury- biochemical markers. Arch Neurol..

[6] Czigner A, Mihály A, Farkas O, Büki A, Krisztin-Péva B, Dobó E (2007). Kinetics of the cellular immune response following closed head injury. Acta Neurochir (Wien)..

[7] Brain Trauma Foundation. Coma guidelines; early indicators of prognosis in severe traumatic brain injury. Assessed on July 18 2019

[8] Chang DC, Cornwell EE, Phillips J, Paradise J, Campbell K (2003). Early leukocytosis in trauma patients: what difference does it make?. Curr Surg..

[9] Sim SK, Lim SL, Lee HK, Liew D, Wong A. Care of severe head injury patients in the Sarawak General Hospital: intensive care unit versus general ward. MJM. 2011:138–41. 22106695

[10] Rimel RW, Giordani B, Barth JT, Boll TJ, Jane JA (1981). Disability caused by minor head injury. Neurosurgery..

[11] Kraus JF, Peek-Asa C, McArthur D (2000). The independent effect of gender on outcomes following traumatic brain injury: a preliminary investigation. Neurosurg Focus..

[12] Susman M, DiRusso SM, Sullivan T, Risucci D, Nealon P, Cuff S (2002). Traumatic brain injury in the elderly: increased mortality and worse functional outcome at discharge despite lower injury severity. J Trauma..

[13] Heiskanen O, Sipponen P (1970). Prognosis of severe brain injury. Acta Neurol Scand..

[14] Marion DW, Carlier PM (1994). Problems with initial Glasgow Coma Scale assessment caused by prehospital treatment of patients with head injuries: results of a national survey. J Trauma..

[15] Marshall LF, Klauber GT, Eisenberg HM (1991). The outcome of severe closed head injury. J Neurosurg..

[16] Van Dongen KJ, Braakman R, Gelpke GJ (1983). The prognostic value of computerized tomography in comatose head-injured patients. J Neurosurg..

[17] Narayan RK, Greenberg RP (1981). Improved confidence of outcome prediction in severe head injury. J Neurosurg..

